# Comparing pharmacists versus allergists in low-risk penicillin allergy delabelling: The Hong Kong Penicillin Allergy Pharmacist Initiative (HK-PAPI)

**DOI:** 10.1016/j.waojou.2024.101003

**Published:** 2024-11-21

**Authors:** James K.Y. Hooi, Marshall C.H. Low, Jonathan C.L. To, Hugo W.F. Mak, Mandy M. Choi, Chris C.P. Tam, Raymond W.M. Mak, Vincent K.C. Wong, Timo C.C. Chan, Andrew W.T. Li, Charlie C.Y. Mak, Valerie Chiang, Gordon K.H. Chu, Jane C.Y. Wong, Philip H. Li

**Affiliations:** a Division of Rheumatology and Clinical Immunology, Department of Medicine, Queen Mary Hospital, The University of Hong Kong, Hong Kong SAR, China; b Department of Pharmacy and Pharmacology, The University of Hong Kong, Hong Kong SAR, China; c Department of Pharmacy, Queen Mary Hospital, Hong Kong SAR, China; d Division of Clinical Immunology, Department of Pathology, Queen Mary Hospital, Hong Kong SAR, China

**Keywords:** Hypersensitivity, Delabelling, Low-risk, Penicillins, Pharmacists

## Abstract

**Background:**

Mislabelled penicillin allergies are associated with a myriad of adverse outcomes and development of anti-microbial resistance. With the overwhelming need for specialist allergy services, pharmacist initiatives such as the Hong Kong Penicillin Allergy Pharmacist Initiative (HK-PAPI) have been advocated. However, evidence of their effectiveness, safety and impact on health-related quality-of-life (HR-QoL) are lacking.

To assess and compare the effectiveness, safety and improvements on HR-QoL of pharmacists vs allergists in a pilot low-risk penicillin allergy delabelling initiative.

**Methods:**

All adult patients referred for low-risk penicillin allergy were randomized and evaluated by either pharmacists or allergists in a 1:3 ratio. Outcomes and changes in Drug Hypersensitivity Quality of Life Questionnaire (DrHy-Q) scores were compared.

**Results:**

Of 323 patients referred, 96.3% (311/323) completed penicillin allergy evaluation (pharmacists: 83 [24.3%] vs allergists: 228 [66.7%]). Overall, 93.6% (291/311) were delabelled with no difference between evaluations by pharmacists and allergists (92.8% vs 93.9%, p = 0.729). There were no severe or systemic reactions in either cohort. Patients evaluated by either pharmacists (43.4 [SD:29.1] to 10.5 [SD:5.93], p < 0.001) or allergists (37.2 [SD:22.2] to 29.1 [SD:22.4], p < 0.001) reported improved HR-QoL as reflected by DrHy-Q scores. However, absolute changes in DrHy-Q scores were significantly greater among patients evaluated by pharmacists compared to those by allergists (−24.6 [SD:25.1] vs −9.19 [SD:13.7], p < 0.001).

**Conclusions:**

Evaluations and delabelling by pharmacists (vs allergists) were comparably effective and safe among patients with low-risk penicillin allergy. Moreover, patients evaluated by pharmacists even reported significantly greater improvements in HR-QoL, highlighting the potential of multidisciplinary allergy initiatives.

## Introduction

Penicillins are the most widely prescribed antibiotics due to their broad range of bactericidal activity, clinical efficacy and overall excellent safety profile.^1^^,^^2^ Unfortunately, penicillins are also the most commonly labelled culprit of drug allergy, with the prevalence of reported penicillin allergy labels estimated to be around 2–13% globally.^3^, ^4^, ^5^, ^6^, ^7^ However, the majority of penicillin allergy labels are found to be incorrect (around 90% mislabelled) with most patients deemed low-risk of genuine allergy upon allergy evaluation.^8^, ^9^, ^10^, ^11^ The impact of penicillin allergy labels is often neglected or underestimated. Inaccurate or unverified allergies are associated with a myriad of adverse outcomes including obligatory use of less effective antibiotic alternatives, increased mortality and hospitalization, higher healthcare costs, and the development of multidrug-resistant microorganisms.^12^, ^13^, ^14^, ^15^ In addition, incorrect penicillin allergy labels can also impair health-related quality-of-life (HR-QoL).^16^ These adverse outcomes may be mitigated by delabelling these incorrect labels.^17^, ^18^, ^19^

Traditionally, allergists evaluate patients with suspected penicillin allergies comprising of history taking, skin tests (ST) and, if negative, drug provocation tests (DPT) – the gold standard to confidently exclude and delabel suspected allergy. However, effective penicillin allergy delabelling has been severely limited due to the severe lack of allergy services and physicians, especially in the Asia Pacific region where the waiting times for allergist consultation or testing were unsatisfactory.^20^, ^21^, ^22^ To overcome this, non-specialist and multi-disciplinary penicillin allergy delabelling initiatives have been advocated in these areas.^23^^,^^24^ Within Asia Pacific countries, it is now recognized and recommended that allergy testing can be independently performed by non-specialists, including trained allied health staff such as nurses and pharmacists. Prior reports have also previously demonstrated the potential of non-physician-directed assessments.^25^, ^26^, ^27^, ^28^, ^29^ As trained professionals with pharmaceutic knowledge and essential members of the healthcare team, pharmacists can potentially play even greater roles in drug allergy services. However, the role of pharmacists in drug allergy has been limited and likely under-utilized, especially in the Asia Pacific region. Furthermore, prior international studies on pharmacist penicillin allergy evaluations have mostly been physician-dependent, cross-sectional or only with indirect comparisons with traditional allergist assessments.^29^, ^30^, ^31^, ^32^, ^33^, ^34^, ^35^ The lack of evidence on their efficacy, safety and longitudinal impact severely limits the greater widespread implementation of pharmacist initiatives.^36^

To overcome this, this study was conducted to assess and compare the effectiveness, safety and improvements in HR-QoL of pharmacists versus allergists in a pilot low-risk penicillin allergy delabelling initiative – the Hong Kong Penicillin Allergy Pharmacist Initiative (HK-PAPI).

## Methods

A territory-wide penicillin allergy delabelling initiative, known as the Hong Kong Drug Allergy Delabelling Initiative (HK-DADI) was established in Hong Kong since 2022.^23^ As defined by HK-DADI consensus and seen in Supplementary Table 1, patients defined as “low risk” can be independently assessed by non-allergists (including trained pharmacists) in Hong Kong. Since 2023, the pilot HK-PAPI started in the Hospital Authority Hong Kong West Cluster and began to run alongside existing allergist penicillin allergy evaluations in accordance with local guidance.^23^ All pharmacists underwent allergy training and were certified to be competent in penicillin evaluation (including performing penicillin allergy skin testing) by accredited Specialists in Immunology & Allergy (accredited by the Hong Kong College of Physicians) at the Hospital Authority Hong Kong West Cluster. All patients gave informed consent. This study was approved by the Institutional Review Board of the University of Hong Kong/Hospital Authority Hong Kong West Cluster.

### Patient recruitment

**Fig. 1:**
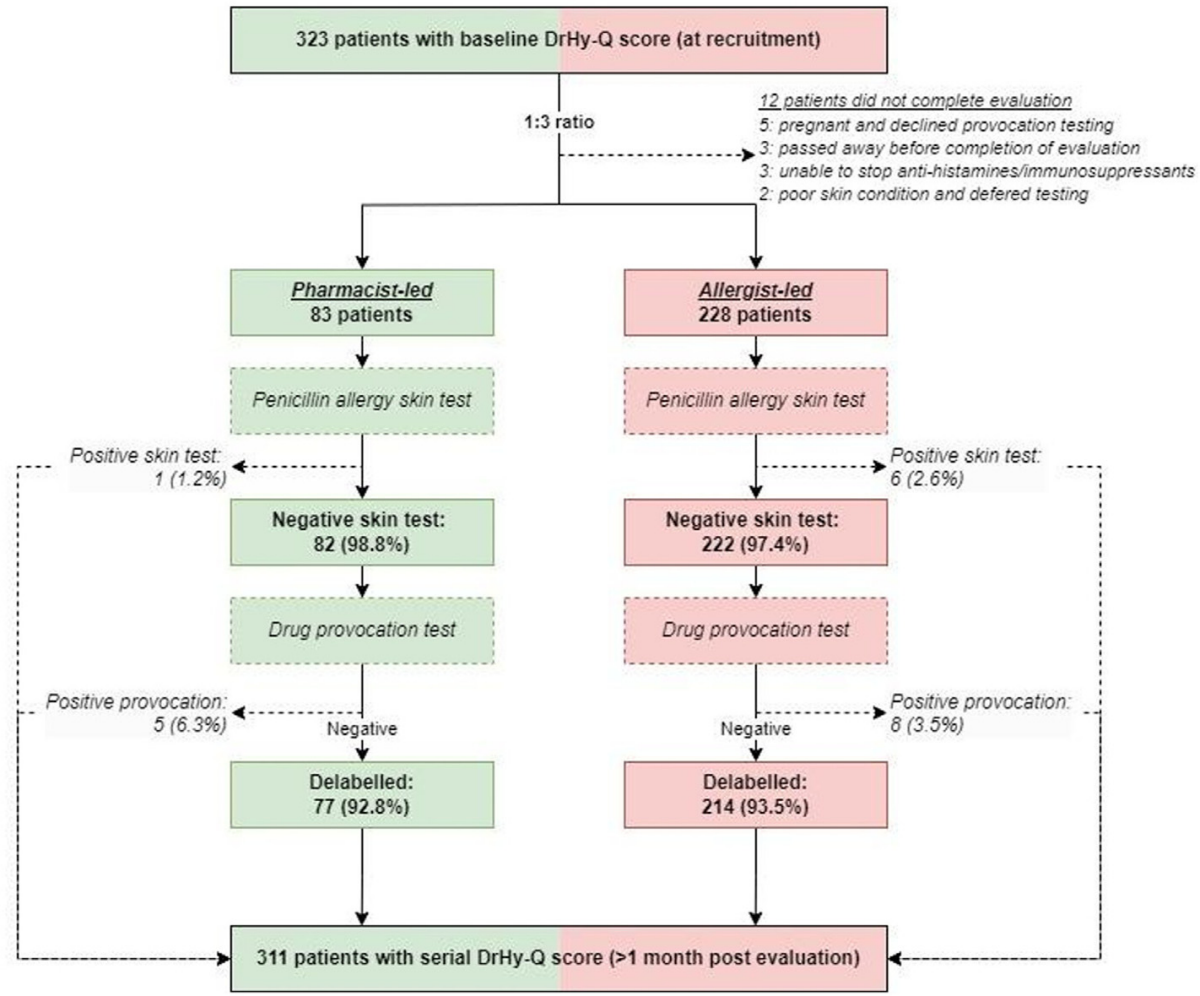
Flowchart of study design and patient recruitment of HK-PAPI.

All referrals for penicillin allergy assessment were preliminarily screened by written or telephone communication with the 6-item Drug Hypersensitivity Quality of Life questionnaires (DrHy-Q) scored as described in previous studies.^19^^,^^27^ The 6-item DrHy-Q, as shown in Supplementary Table 2, consisted of 6 questions, with a total score of 0–100, and patients with a lower score indicated a better HR-QoL. All patients who met “low risk” criteria as per HK-DADI criteria were allocated for evaluation by either trained pharmacists or allergists (standard of care). As this was a pilot study with limited pharmacist manpower, cases were assigned at random (by random number generator) in a 1:3 (pharmacists: allergists) ratio from January to December 2023. A flowchart of study design and patient recruitment is summarized in Fig. 1.

### Patient assessment and allergy testing

Patients evaluated by either pharmacists or allergists were interviewed and counselled on the indications, procedures and risks of penicillin allergy testing as per HK-DADI recommendations.^23^ Patients were counselled on the low pre-test probability of genuine penicillin allergy (based on history and meeting low-risk criteria) and consented to further testing due to favourable benefit-risk ratio. All patients who agreed to testing and provided written consent were offered ST, with or without DPT, by either trained pharmacists or allergists. ST and DPT protocols were the same and performed independently by either pharmacists or allergists. Resuscitation facilities, appropriate monitoring equipment and physicians were available throughout drug allergy testing in both the allergist and pharmacist groups.^23^

Upon initial evaluation by pharmacist or allergist, low risk patients were triaged into immediate- and non-immediate-type reactions, and ST (skin prick [SPT] and intradermal tests [IDT]) were performed with benzylpenicilloyl polylysine (0.04 mg/mL), sodium benzylpenilloate (0.5 mg/mL), benzylpenicillin (6 mg/mL), and amoxicillin (20 mg/mL). When clinically indicated or when it was the index penicillin, selected patients would also undergo additional ST according to European Network and European Academy of Allergy and Clinical Immunology recommendations.^37^ Results of SPT and IDT were read at 15 min. A positive SPT was defined as a wheal 3 mm diameter or greater. A positive IDT was defined as a wheal expansion of 3 mm or greater from the original bleb. Patients with positive ST were deemed likely to be genuinely allergic and did not proceed with subsequent DPT. Patients with negative ST proceeded with DPT with the same penicillin according to their index reaction, and placebos were not used. If the index penicillin was unknown, the DPT was performed with amoxicillin in accordance to local recommendations (three-step protocol, eg, 10%, 30%, 60% of maximum single unit dose, in 30-min intervals).^23^ Oral formulations with full adult doses were chosen, when available.

Patients were interviewed 1 month after testing for review with DrHy-Q repeated. All evaluations that were completed between January and December 2023 were included for analysis. All demographic, clinical and allergy outcomes were retrieved and anonymized, then compared between pharmacist and allergist evaluations.

### Statistical analysis

All statistics were analysed using IBM SPSS Statistics 28.0 (IBM Corp., Armonk, NY, USA). Baseline demographic and clinical data between the pharmacist and allergist cohorts are compared using X^2^ statistics and student's t-test. Values were presented as numbers (percentages) as appropriate. Comparisons of delabelling rates between allergist and pharmacist cohorts were analysed using X^2^ statistics. Changes in DrHy-Q scores pre- and post-intervention for allergist and pharmacist cohorts were compared using Wilcoxon signed-rank test while the differences in DrHy-Q scores changes between both pathways were compared using Mann-Whitney *U* test. Values of DrHy-Q scores are presented with mean (Standard Deviation [SD]) as appropriate. Two-sided P less than 00.05 was considered significant in all statistical tests. Figures of DrHy-Q scores were constructed using Plotly Chart Studio (Plotly Technologies Inc., Montréal, QC, Canada).

## Results

Within the 1-year study period, a total of 323 patients with low-risk penicillin allergy were referred, of which 96.3% (311/323) completed evaluation (pharmacists: 83 [24.3%] vs allergists: 228 [66.7%]) (Fig. 1). The median age was 58.0 (IQR: 25.5) years and the male:female ratio was 2:1. There were no significant differences in the baseline demographics, clinical features pertaining to suspected penicillin allergy and baseline DrHy-Q scores, as detailed in Table 1.

**Table 1:** Baseline demographic & clinical characteristics of pharmacist vs allergist cohorts

Parameters	All (n = 311)	Pharmacist cohort (n = 83)	Allergist cohort (n = 228)	P value
Female sex; n (%)	205 (65.9)	55 (71.1)	146 (64.0)	0.246
Age; median years (IQR)	58.0 (25.5)	60 (31.5)	57.0 (24.0)	0.607
Duration of penicillin allergy label				0.201
- *≤5 years*	*237 (76.2)*	*24 (28.9)*	*50 (21.9)*	
- *>5 years*	*74 (23.8)*	*59 (71.1)*	*178 (78.1)*	
Type of reaction; n (%)				0.219
- *Immediate*	*73 (23.5)*	*21 (25.3)*	*52 (22.8)*	
- *Non-immediate*	*120 (38.6)*	*37 (44.6)*	*83 (36.4)*	
- *Unknown*	*118 (37.9)*	*25 (30.1)*	*93 (40.8)*	
Concomitant drug allergy labels; n (%)	149 (47.9)	36 (43.4)	113 (49.6)	0.334
Baseline DrHy-Q scores (SD)	38.9 (24.4)	37.2 (22.2)	43.4 (29.1)	0.173

### Delabelling rate of 93%, no difference between pharmacists and allergists

As shown in Table 2, 93.6% (291/311) patients were successfully delabelled of previous mislabelled penicillin allergy, with no differences in delabelling rates between pharmacists and allergists (92.8% vs 93.9%, p = 0.729). Only 1 (1.2%) and 5 (6.3%) in the pharmacist cohort, compared to 6 (2.6%) and 8 (3.6%) in the allergist cohort, had positive ST and DPT, respectively.

**Table 2:** Outcomes of allergy testing in pharmacist vs allergist cohorts

Parameters	All (n = 311)	Pharmacists cohort (n = 83)	Allergists cohort (n = 228)	P value
Positive skin tests; n (%)	7 (2.25)	1 (1.20)	6 (2.63)	0.680
Positive drug provocation tests; n (%)	13 (4.18)	5 (6.02)	8 (3.51)	0.343
Delabelled penicillin allergy; n (%)	291 (93.6)	77 (92.8)	214 (93.9)	0.729

### Similar ST performance and safety between pharmacists and allergists

The negative predictive value of ST by pharmacists and allergists was therefore similar at 93.9% and 96.4%, respectively. No patients in either cohort experienced systemic or severe reactions from ST or DPT. Detailed medical history and outcomes of patients with positive ST or DPT results are summarized in Table 3.

**Table 3:** Details of outcome and results of patients with positive ST/DPT

Cohort	Case no	Age	Sex	Index Drug	Reaction type	ST result	DPT result
Pharmacist	4	56	F	Ampicillin	Unknown	Neg	Pos
Pharmacist	29	59	F	Ampicillin & amoxicillin	Delayed	Neg	Pos
Pharmacist	55	59	F	Amoxicillin-clavulanate	Delayed	Neg	Pos
Pharmacist	57	79	M	Amoxicillin-clavulanate	Delayed	Pos	ND
Pharmacist	62	32	F	Amoxicillin-clavulanate	Immediate	Neg	Pos
Pharmacist	97	36	F	Amoxicillin-clavulanate	Delayed	Neg	Pos
Allergist	25	53	F	Amoxicillin	Immediate	Neg	Pos
Allergist	31	37	M	Penicillin V	Unknown	Pos	Neg
Allergist	54	53	M	Penicillin V	Unknown	Pos	Neg
Allergist	77	37	F	Amoxicillin	Delayed	Neg	Pos
Allergist	124	81	F	Unknown penicillin	Immediate	Neg	Pos
Allergist	135	77	F	Unknown penicillin	Unknown	Pos	Neg
Allergist	139	68	F	Amoxicillin-clavulanate	Delayed	Neg	Pos
Allergist	145	70	F	Unknown penicillin	Delayed	Neg	Pos
Allergist	160	59	F	Amoxicillin	Delayed	Pos	Neg
Allergist	176	47	F	Piperacillin-tazobactam	Unknown	Neg	Pos
Allergist	185	73	M	Amoxicillin-clavulanate	Immediate	Neg	Pos
Allergist	197	55	M	Amoxicillin	Unknown	Neg	Pos
Allergist	240	37	F	Amoxicillin	Delayed	Pos	Neg
Allergist	261	41	M	Amoxicillin-clavulanate	Delayed	Pos	Neg

Pos: positive. Neg: negative. ND: Not done

### Serial improvements in HR-QoL irrespective of allergy evaluation outcome

Overall, there were serial improvements in HR-QoL as reflected by reduced DrHy-Q scores among all patients (38.9 [SD:24.4] to 26.3 [SD:23.5]; change: −12.7 [SD:20.1] p < 0.001). Subgroup analysis showed no difference in HR-QoL improvements regardless of evaluation outcome (positive ST/DPT vs negative DPT: −41.7 [SD:30.2] vs −23.3 [SD:24.4], p = 0.127).

### Significantly greater improvements in HR-QoL in pharmacist vs allergist cohort

**Fig. 2:**
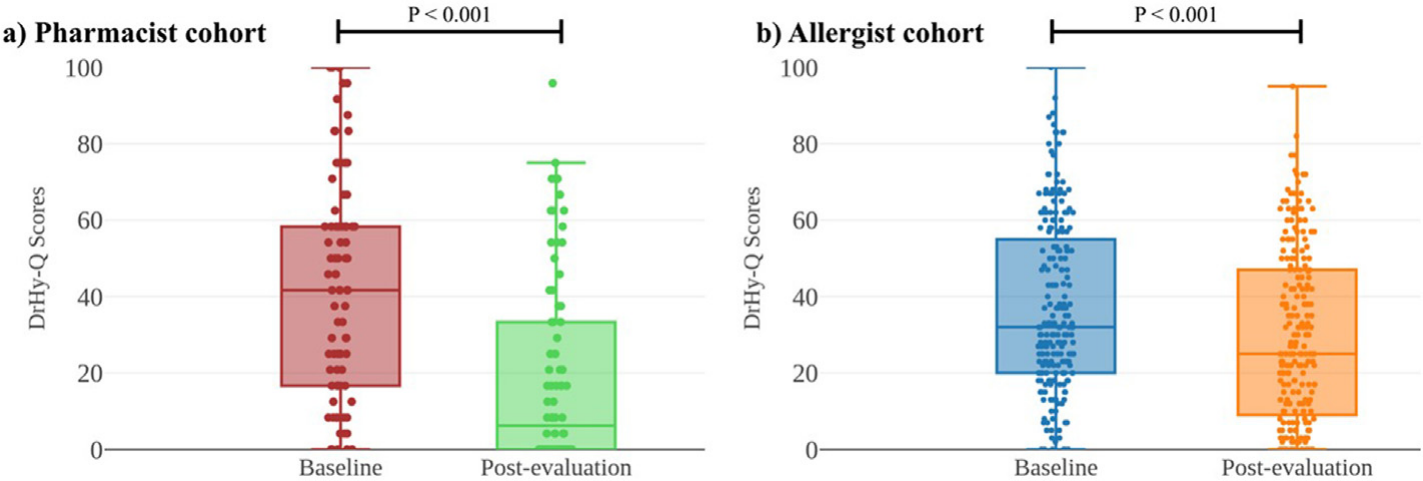
Box and whisker plots of baseline vs post-evaluation DrHy-Q scores of a) pharmacist and b) allergist cohort.

Regardless of evaluation by pharmacists or allergists, patients reported significantly improved HR-QoL as reflected by DrHy-Q scores in both groups (Fig. 2). However, absolute changes in DrHy-Q scores were significantly greater among patients who were evaluated by pharmacists compared to allergists (−24.6 [SD:25.1] vs −9.19 [SD:13.7], p < 0.001).

## Discussion

Although other pharmacist-led penicillin allergy services have been previously described, we report the first direct comparison between evaluation performed by pharmacists vs allergists, especially in Asia. Earlier reports on pharmacist initiatives were primarily observational with only indirect measures of outcomes. Moreover, our study uniquely investigated the longitudinal impact of pharmacist services on patient HR-QoL. Most previous studies have only primarily focused on the imminent effects of drug allergies or restrictions in drug allergy labels. However, the impact of delabelling incorrect drug allergies on overall HR-QoL (ie, beyond just the imminent need of medication use) using patient reported outcome measures has been a growing area of active research.^16^^,^^18^ This is especially important as patients with history of drug allergy are at risk of developing various psychiatric and mental comorbidities. For example, drug induced anaphylaxis can cause long-term psychological disorders such as depression and anxiety.^38^ Previous Chinese studies also demonstrated increased risk of psychiatric disorders among patients with history of severe cutaneous adverse reactions from drug allergy.^39^ Nevertheless, it is often important to re-evaluate these presumed drug allergies as many are mislabelled or patients may have lost sensitivity over time.^40^ Our study therefore highlights the additional and previously under-recognized benefits of multidisciplinary drug allergy initiatives.

Overall, patients evaluated by either pharmacists or allergists demonstrated significant improvements in HR-QoL as measured by serial DrHy-Q scores. Consistent with previous experience, we noted longitudinal improvements in HR-QoL regardless of evaluation outcomes (ie, positive or negative ST or DPT).^16^ In fact, among the minority (6 patients) whom had positive allergy tests, their change in DrHy-Q scores were not significantly different to those of delabelled patients. We postulate that the benefits of undergoing penicillin evaluation by trained professionals - including comprehensive review of medical histories and detailed counselling as well as patient education on future medication use – would benefit HR-QoL regardless of delabelling outcome. Through conveying greater details of their penicillin allergy and subsequent delabelling, patient anxiety, stress and concerns may also be further alleviated. Due to the small sample size of patients with positive allergy tests, these findings will need to be validated by multi-centre collaborative studies investigating serial changes in HR-QoL in the future.

In addition, patients evaluated by pharmacists demonstrated significantly greater reductions in serial DrHy-Q scores. As trained professionals with pharmaceutic knowledge, pharmacists are well equipped for patient education and counselling. Differences and advantages of pharmacists in patient communication and education may, at least partially, explain the superior improvements in DrHy-Q scores among patients evaluated by pharmacists vs allergists. Other studies demonstrating the significant impact of pharmacist initiatives on patient HR-QoL beyond drug allergy (including patients with heart failure, asthma, diabetes mellitus and epilepsy) have similarly been reported.^41^, ^42^, ^43^, ^44^ Further dedicated studies comparing longitudinal outcomes of drug allergy HR-QoL are needed to confirm consistency and external validity of our findings.

Consistent with prior studies of low-risk penicillin allergy, the role of ST prior to DPT remains questionable.^19^^,^^27^ Only 1 single patient in the pharmacist cohort and less than 3% of the allergist cohort had positive ST. Although ST have traditionally been advocated (especially for suspected IgE-mediated reactions), their low reproducibility and variable positive predictive value diminishes its value among low-risk cohorts - where the pre-test probability of genuine penicillin allergy is already low.^45^, ^46^, ^47^ Other reported pharmacist-led penicillin allergy services have skipped ST in their practice and report similar safety.^48^, ^49^, ^50^ Following findings from HK-PAPI, our pharmacists have also started to conduct direct DPT among patients with low-risk penicillin allergy since 2024. A prospective study investigating the reductions in overall costs and waiting time, as well possible additional improvements in delabelling rate and HR-QoL, is currently underway.

Penicillin allergy is an ever-growing concern in face of increasingly multidrug-resistant organisms, making accurate but safe allergy assessments crucial to guide antibiotic stewardship. Furthermore, there has been increasing recognition of the importance of proper drug allergy evaluation especially following the initial fears of vaccine-related events during the COVID19 pandemic.^51^, ^52^, ^53^, ^54^, ^55^ In fact, this novel penicillin allergy pharmacist initiative, along with other multidisciplinary allergy initiatives, was initially established out of necessity to overcome the unmet needs of drug allergy services in Hong Kong.^21^^,^^56^ Serendipitously, non-allergist-led penicillin allergy delabelling services have not only consistently comparable outcomes and safety among low-risk penicillin allergy patients, but also improved waiting times and rate of future penicillin use.^19^^,^^27^ Reducing the burden of low-risk patients could also allow allergists to dedicate resources to high-risk or complex cases, and reduce overall waiting time for allergy services.^20^^,^^21^ However, this could only be achieved with adequate training and support from allergists as well as clear definitions of “low-risk patients”.^23^^,^^24^ A comprehensive training programme, including the entire medical team in both procedural and safety training (eg, simulation training for potential anaphylaxis) and accreditation is also immensely important.^57^ This highlights the importance of prior risk stratification, as well as close collaboration between allergists and non-specialists.

Prior successes in such multidisciplinary collaborations have translated to the establishment of multiple territory-wide non-specialist penicillin allergy clinics, as well as incorporation of such strategies in international recommendations.^19^^,^^24^ However, given the dire shortage of physicians and nurses in our locality, further collaborations with pharmacists and other allied health professionals are important to address the unmet local and regional needs in drug allergy services.^22^^,^^23^

There were numerous limitations in this study. Firstly, as a 1-year pilot study with limited manpower available, our sample size was limited and therefore patients were randomized in a 1:3 ratio between pharmacists and allergists. This ratio was decided on practical need to balance waiting times and available resources. Secondly, all pharmacists were trained certified by accredited specialists in Immunology & Allergy and only conducted evaluations in a single tertiary referral centre in Hong Kong. Thirdly, patients with positive ST did not proceed with DPT despite being low risk, and we could not exclude the possibility of false positive ST. Lastly, we only recruited patients actively referred by clinicians for penicillin allergy delabelling, leading to a possibility of ascertainment bias. Hence, certain outcomes such as improved HR-QoL may not be universally valid. These limitations highlight the importance of prospective, multi-centre validation studies in the future.

In conclusion, we demonstrated that evaluations and delabelling by pharmacists (vs allergists) were comparably effective and safe among patients with low-risk penicillin allergy. Moreover, patients evaluated by pharmacists reported even greater improvements in HR-QoL. We advocate for wider adoption of multi-disciplinary collaborations to overcome the ever-increasing burden of the drug allergy pandemic.

## Abbreviations

DPT, Drug provocation tests; DrHy-Q, Drug Hypersensitivity Quality of Life Questionnaire; HK-DADI, Hong Kong Drug Allergy Delabelling Initiative; HK-PAPI, Hong Kong Penicillin Allergy Pharmacist Initiative; HR-QoL, Health-related quality-of-life; IDT, Intradermal test; SPT, Skin prick test; ST, Skin tests.

## Availability of data and materials

Data available from the corresponding author upon reasonable request.

## Author contributions

Conception and design of study: PHL; Acquisition of data: MMC, CCPT, RWMM, VKCW, TCCC, AWTL, CCYM, VC, GC, JCYW; Analysis and interpretation of data: MCHL, JCLT, HWFM; Drafting and revising the article: JKYH, PHL; Final approval: All authors.

## Authors’ consent for publication in WAO

All authors consent for publication in WAO.

## Ethics

All patients gave informed consent. This study was approved by the Institutional Review Board of the Hong Kong West Cluster/The University of Hong Kong.

## Financial support

There was no funding for this study.

## Declaration of competing interest

The authors report no competing interests.
