# Supplementary material for: Comparing pharmacists versus allergists in low-risk penicillin allergy delabelling: The Hong Kong Penicillin Allergy Pharmacist Initiative (HK-PAPI)
Source: World Allergy Organ J. 2024 Nov 21;17(12):101003. doi: 10.1016/j.waojou.2024.101003 (PMC11617727; doi:10.1016/j.waojou.2024.101003)
Supplement: Multimedia component 1 [file mmc1.docx]

**Supplementary Table 1: Risk stratification of penicillin allergy according to the Hong Kong Drug Allergy Delabelling Initiative**

| **High Risk: Any of the following** | |  |
| --- | --- | --- |
| - Anaphylaxis - Hypotension (Dizziness / syncope) - Respiratory compromise (bronchospasm, wheeze, desaturation) - Urticaria or angioedema (<1 hour of penicillin exposure) | - Documented SCAR (TEN-SJS / DRESS / AGEP) - Mucosal involvement - Eosinophillia - Internal organ involvement (e.g. cytopenias, liver/renal dysfunction) - Drug-induced autoimmune disease or vasculitis |  |
| **Medium Risk: Absence of high risk-features, and any of the following:** | |  |
| - Flushing or redness - Nasal symptoms - Subjective chest tightness or difficulty in breathing/swallowing - Throat tightness or hoarseness of voice - Non-urticarial rash | - Urticaria or angioedema (>1 hour of penicillin exposure) - Blistering and/or skin desquamation - Lymphadenopathy - Fever (after penicillin exposure) |  |
| **Low Risk: Absence of high or medium-risk features, and any of the following:** | |  |
| - Unknown history and > 1 year ago - Family history without personal history - Previously told allergy test positive but no history of reaction - Other non-betalacktam allergies only | - Isolated gastrointestinal upset - Headache - Fatigue - Chills or rigor - Itching only, without rash |  |

AGEP, Acute generalized exanthematous pustulosis; DRESS, drug rash with eosinophilia and systemic symptoms; SCAR, severe cutaneous adverse reaction; TENS/SJS, toxic epidermal necrolysis, Steven-Johnson syndrome.

**Supplementary Table 2: 6-item Drug Hypersensitivity Quality of Life Questionnaire (DrHy-Q)**

For each of the following questions, please tick the box that best describes your situation.

1 = Not at all, 2 = Little, 3 = Somewhat, 4 = Much, 5 = Very much

|  | Not at all | Little | Somewhat | Much | Very Much |
| --- | --- | --- | --- | --- | --- |
| 1. I feel frightened due to my problem of allergy reaction. | 1 | 2 | 3 | 4 | 5 |
| 2. The problem of adverse reaction to drugs affects my life. | 1 | 2 | 3 | 4 | 5 |
| 3. The fact that I cannot use medication safely made me feel different from others. | 1 | 2 | 3 | 4 | 5 |
| 4. I feel anxious due to my problem of allergy reaction. | 1 | 2 | 3 | 4 | 5 |
| 5. I feel anguished due to my problem of allergy reaction. | 1 | 2 | 3 | 4 | 5 |
| 6. The idea of taking a medicine makes me feel anxious. | 1 | 2 | 3 | 4 | 5 |
|  | Total score: _________ | | | | |
